# Pseudo Optimization of E-Nose Data Using Region Selection with Feature Feedback Based on Regularized Linear Discriminant Analysis

**DOI:** 10.3390/s150100656

**Published:** 2014-12-31

**Authors:** Gu-Min Jeong, Nguyen Trong Nghia, Sang-Il Choi

**Affiliations:** 1 Electrical Engineering, Kookmin University, 861-1, Jeongeung-dong, Songbuk-gu, Seoul 136-702, Korea; E-Mails: gm1004@kookmin.ac.kr (G.-M.J.); nghiavp07@gmail.com (N.T.N.); 2 Department of Computer Science and Engineering, Dankook University, 126, Jukjeon-dong, Suji-gu, Yongin-si, Gyeonggi-do 448-701, Korea

**Keywords:** e-nose system, vapor classification, feature feedback, discriminant feature

## Abstract

In this paper, we present a pseudo optimization method for electronic nose (e-nose) data using region selection with feature feedback based on regularized linear discriminant analysis (R-LDA) to enhance the performance and cost functions of an e-nose system. To implement cost- and performance-effective e-nose systems, the number of channels, sampling time and sensing time of the e-nose must be considered. We propose a method to select both important channels and an important time-horizon by analyzing e-nose sensor data. By extending previous feature feedback results, we obtain a two-dimensional discriminant information map consisting of channels and time units by reverse mapping the feature space to the data space based on R-LDA. The discriminant information map enables optimal channels and time units to be heuristically selected to improve the performance and cost functions. The efficacy of the proposed method is demonstrated experimentally for different volatile organic compounds. In particular, our method is both cost and performance effective for the real implementation of e-nose systems.

## 1. Introduction

Electronic nose (e-nose) systems classify different odors, chemical components and vapors with sensor arrays and have been widely studied and developed [1–18]. In particular, diverse studies have researched the materials, chemical reactions, packaging, sensor arrays, pattern recognition and embedded system designs of e-nose systems.

Pattern recognition and data mining processes are essential for e-nose systems. Since two-dimensional data are obtained from numerous sensor channels with different characteristics, it is important to utilize efficient and suitable pattern-recognition methods [19–29]. Processing e-nose data effectively can potentially improve the performance of the designed hardware. Moreover, the results can be used to design additional sensors.

Numerous studies have applied pattern-recognition methods, such as feature extraction or selection, to e-nose systems [8–18]. In [11], template matching was adopted to classify e-nose data into two-dimensional image form. In [12–15], linear discriminant analysis (LDA), support vector machine (SVM) and relative vector machine (RVM) were used for classification. Various optimization-like techniques have also been proposed to reduce the number of sensor arrays [16,17] and the processing time-horizon [18]. In [17], the rough set-based optimization technique was proposed to select sensor channels. In [16,18], feature feedback-based pattern-recognition methods were proposed for e-nose systems. In [24], feature feedback is introduced as a data refinement technique to reduce the redundancy of a high-dimensional face image dataset. For the e-nose dataset used in our paper, by reverse mapping from the feature space to the original data space, using principle component analysis (PCA) and LDA (PCA + LDA), channel selection [16] and time-horizon selection [18] can be achieved. By retaining the important parts of the original data and discarding redundant data, the sensor array was further optimized, and the classification process was made more efficient; specifically, the classification rate, processing time, memory size, *etc.*, demonstrated that the recognition performance was preserved or slightly improved.

We present a pseudo optimization method for e-nose data using feature feedback based on regularized LDA (R-LDA) [28,29] to enhance the performance and cost functions of the e-nose system. Since R-LDA [20] outperforms PCA + LDA in face recognition problems, we expect that the feature feedback using R-LDA will outperform that using PCA + LDA [16,18].

By extending previous feature feedback results [16,18], we obtain a two-dimensional discriminant information map, which is subsequently used to implement a region-based data selection method. In the two-dimensional map, each rectangular region consists of continuous rows and columns that correspond to continuous sensor channels and time units, respectively. In this scheme, important data are defined based on the region from which data are selected from the two-dimensional map; the sensor channels and time units that form the selected region are considered important data. This important information facilitates the improvement of the performance and cost functions. Experimental results for different volatile organic compounds [11] show that our method can classify data better than other existing methods. Furthermore, our method is both cost and performance effective for the real implementation of e-nose systems.

This paper is organized as follows. In Section 2, we review existing literature on R-LDA-based feature feedback. We present region selection methods in Section 3 and present our experimental results in Section 4. In Section 5, we state our concluding remarks.

## 2. Feature Feedback Using R-LDA

### 2.1. Feature Feedback

**Figure 1.**
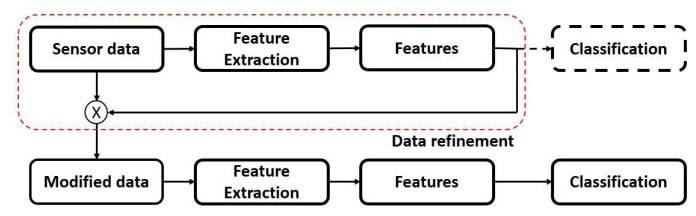
Concept of using feature feedback as a data-refinement method in a classification system.

In [24], feature feedback is proposed as a data pre-processing algorithm to identify important and eliminate redundant data in training and test sets. To reduce the dimension of input data, feature feedback uses several common feature extraction techniques, such as PCA, LDA or R-LDA, to create a feature mask that is then used as a reverse mapping from the feature space to the input space. Figure 1 illustrates the principle idea of utilizing feature feedback compared with other feature extraction methods in a classification system. Instead of directly using the extracted features for the classification, in feature feedback, these features are used to revert to the original data as a data-refinement process.

**Figure 2.**
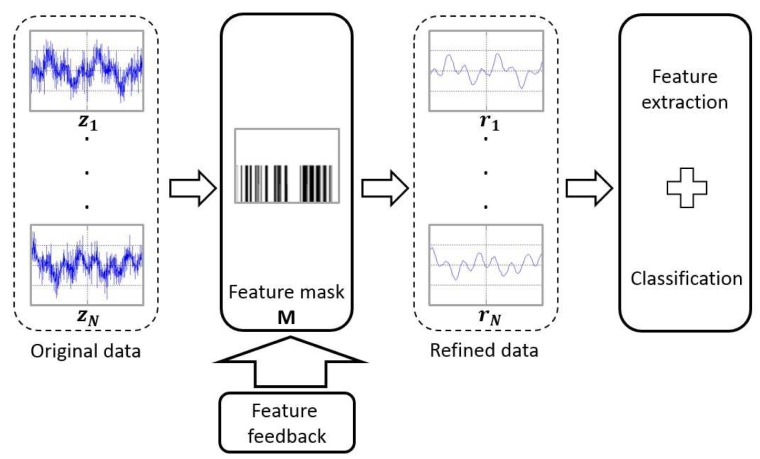
Vapor classification based on feature feedback.

To accomplish this, as shown in Figure 2, feature feedback uses these extracted features to create a feature mask and multiplies this mask to the original data. The feature mask obtained from the feature feedback stage is a binary mask in which “1” elements indicate important pieces of the mask and the “0” elements represent unimportant parts. Consequently, the pixels in the input samples that are important for the classification can be selected in this form of feature mask.

### 2.2. Regularized Linear Discriminant Analysis

In this section, we briefly introduce the concept of R-LDA from the viewpoint of improving the LDA method [19]. R-LDA attempts to solve the small sample size (SSS) problem.

Let $Z={\left\{Z_{i}\right\}}_{i=1}^{C}$ be a training set consisting of *C* classes **Z***_i_*. Each class **Z***_i_* consists of *C_i_* samples ${\left\{z_{ij}\right\}}_{j=1}^{C_{i}}$. Overall, a total of $N=\sum_{i=1}^{C}C_{i}$samples are available. For convenience, each sample is represented as a *J*-dimensional matrix, where (*J* = *I_x_* × *I_h_*). The lexicographic ordering operation of LDA locates the set of feature vectors (Fisherfaces), denoted by ${\left\{W_{m}\right\}}_{m=1}^{M}$, which are used to construct the feature space for classification. LDA performs dimensionality reduction, while preserving as much of the class discriminant information as possible. This is achieved by simultaneously maximizing the determinant of the between-class scatter matrix and minimizing the determinant of the within-class scatter matrix. The objective function of LDA can be written as follows:

(1) $$W=\arg\underset{W}{\max}\frac{\left|W^{T}S_{B}W\right|}{\left|W^{T}S_{W}W\right|},W=\left[W_{1},W_{2},\ldots ,W_{M}\right],W_{m}\in {\text{R}}^{J},$$

where **S***_B_* and **S***_W_* are the between and within-class scatter matrices, respectively, defined as follows:

(2) $${\text{S}}_{B}=\sum_{i=1}^{C}N_{i}\left(\mu_{i}-\mu \right){\left(\mu_{i}-\mu \right)}^{T}$$

(3) $${\text{S}}_{W}=\sum_{i=1}^{C}\sum_{x_{k}\in c_{i}}^{C_{i}}\left(x_{k}-\mu_{i}\right){\left(x_{k}-\mu_{i}\right)}^{T}$$

here, $\mu_{i}=\frac{1}{C_{i}}\sum_{j=1}^{C_{j}}z_{ij}$ and *μ* are the mean of the class **Z***_i_* and entire training data, respectively. The optimization problem in Equation (1) is equivalent to the following generalized eigenvalue problem,

(4) $${\text{S}}_{B}W_{m}=\lambda_{m}{\text{S}}_{W}W_{m},m=1,\ldots ,M.$$

The PCA + LDA method attempts to solve the SSS problem by performing PCA [23] before LDA, which results in **S***_W_* being non-singular. However, since the PCA step may discard dimensions that contain important discriminative information, the PCA + LDA method does not give the best solution to the SSS problem. To overcome this problem, R-LDA was developed [20]. R-LDA is the extended version of LDA, which aims to solve the SSS problem. The regularized Fisher's criterion can be expressed as follows:

(5) $$\text{W}=\arg\underset{W}{\max}\frac{\left|W^{T}S_{B}W\right|}{\left|\eta \left(W^{T}S_{B}W\right)+W^{T}S_{W}W\right|},$$

where 0 ≤ *η* ≤ 1 is a regularization parameter. The proof of the equivalence between Equations (1) and (5) can be found in [20].

The scatter matrices and objective functions for PCA, LDA and R-LDA are shown in Table 1. In Table 1, the columns of $W^{F}=\left[w_{1}^{F}w_{2}^{F}‥w_{n\prime }^{F}\right]$, where *F* ∈ {*P*, *L*, *R*}, are the projection vectors. These vectors are used to represent the sample **x***_k_* as a low-dimensional feature vector **y***_k_* = (*W^F^*)*^T^***x***_k_*, where *F* ∈ {*P*, *L*, *R*}, in the *n′*-dimensional feature space.

### 2.3. Feature Feedback Using R-LDA

In [28,29], a basic form of R-LDA-based feature feedback is introduced. To evaluate the relative importance of the information in each variable for classification, the relationship between the basis of the feature space and the input variables are analyzed. After the useful features from the training data are extracted using R-LDA, a feature mask from the feature-related region is constructed. This is then used to refine the input data, including both the test and training sets. Since the R-LDA method has the ability to extract significant features for classification, the feedback step using the feature mask can effectively identify important regions, as well as eliminate redundant regions from the input data. Consequently, the classification performance of feature feedback based on the R-LDA method is expected to be better.

**Figure 3.**
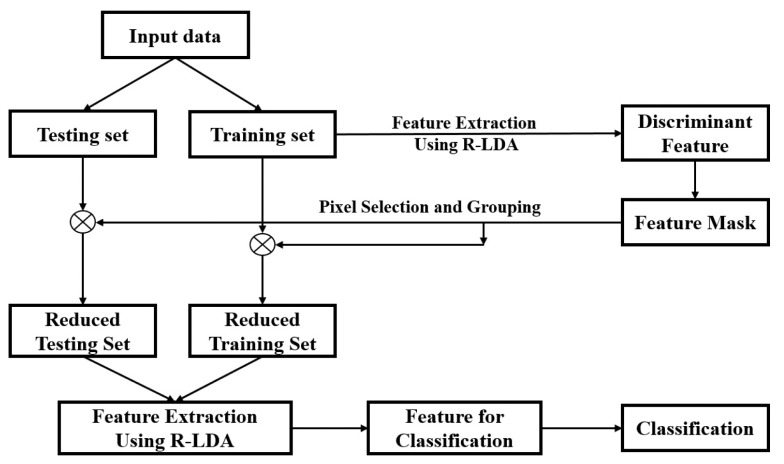
Overall procedure of the R-LDA-based feature feedback.

All of the necessary steps regarding the experiment are shown in Figure 3. The overview procedure is as follows:

- Step 1: The discriminant features used in the feature feedback stage are extracted using R-LDA. Since the projection vectors corresponding to large eigenvalues are more significant feature bases, the first *n_f_* projection vectors with large eigenvalues are selected to use for feature feedback.
- Step 2: A feature mask is constructed by summing *n_f_* projection vectors extracted in Step 1. In the feature mask, **N** elements with large values are set to one, while the remaining elements are set to zero, *i.e.*, the final mask from R-LDA contains only one and zero elements.
- Step 3: The input data are refined using the final mask. The elements in the input data corresponding to one in the final mask are selected and utilized for classification, and the remaining elements in the input data are eliminated.

## 3. Channel and Time-Horizon Selection Using Feature Feedback with R-LDA

**Figure 4.**
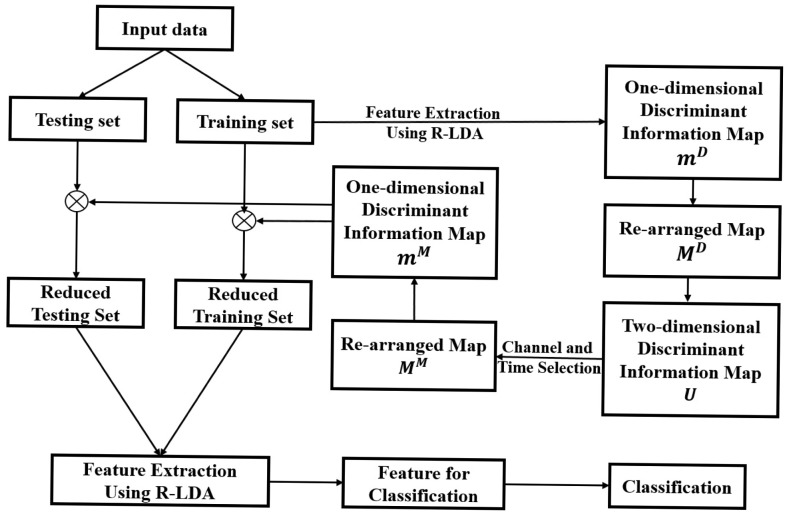
Overall procedure of the proposed method.

In this section, we present a method to extract the important data from a two-dimensional discriminant information map for classification. In [18], a one-dimensional discriminant information map, considering only the time-horizon dimension, is proposed to implement the time-horizon data selection method. On the other hand, the channel selection method in [16] considers only the channel dimension for the implementation of data selection. Since the region selection method in this paper considers both channel and time-horizon dimensions for data selection, a corresponding two-dimensional discriminant information map is created to implement the data selection process. Our method consists of two stages: In the first stage, we derive a two-dimensional discriminant information map using feature feedback based on R-LDA. In the second stage, the channels and time-horizons are selected simultaneously based on the two-dimensional map for classification. The procedure of our channel and time-horizon selection method is shown in Figure 4.

### 3.1. Two-Dimensional Discriminant Information Map for Channels and Time Units

We first measure the distribution of the discriminant information in the data sample by using the feature feedback [18,24]. We then construct a two-dimensional discriminant information map **M***^D^*, which is used as a reference for selecting the channel and time section.

The amount of discriminant information in each element of the data samples is based on projection vectors of R-LDA, $w_{l}^{R}$, where *l* = 1,…, *n_f_*. For each projection vector $w_{l}^{R}$, the magnitude of $w_{li}^{R}$ reflects the amount of discriminant information in the data sample. Therefore, we construct a map $m_{l}^{R}$ for each $w_{l}^{R}$ representing the distribution of discriminant information in the data sample. We then merge $m_{l}^{R}\text{s}$, *l* = 1,…, *n_f_* to obtain a single map **m***^D^*. Each value of $m_{i}^{D}$, *i* = 1, .., *J*, of **m***^D^* indicates the relative amount of discriminant information in element **x***_ki_* of **x***_k_* = [*x_k_*_1_, ‥, *x_kJ_*]*^T^*. For data reduction and normalization, we replace the values of the *N* largest elements in $m_{i}^{D}\text{s}$ with one and set the remaining entries to zero.

**Figure 5.**
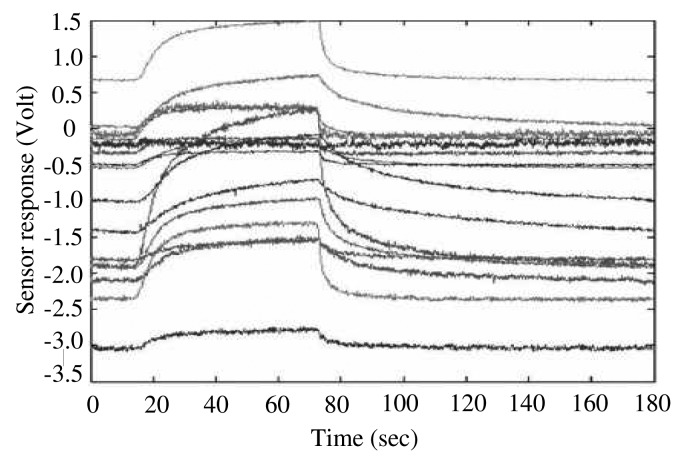
Typical time-responses of 16-channel sensor array with respect to the inflow of toluene.

In our e-nose system, we use a gas sensor array chip consisting of 16 separate channels to collect vapor data samples [16]. Each data sample is acquired through 16 channels over 2000 time points ranging from 0 to 2 s. In this scheme, one data sample is represented by a vector **x***_k_* ∈ ℝ^32000^ in 32,000-dimensional input space. The typical multi-sensor time-response of the toluene vapor [16] is shown in Figure 5. The discriminant information map **m***^D^* is represented by a 32,000-dimensional vector, similar to the input space and projection vectors produced by R-LDA.

After several data processing steps, the distribution of the one-dimensional discriminant information map **m***^D^* can be observed according to the channel and time-horizon. First, the map **m***^D^* is re-arranged as a 16 × 2000 matrix **M***^D^*. Each row of **M***^D^* represents the data of each channel through 2000 time points, and each column represents the data in each time point of the 16 channels. After the transformation, we divide the time-horizon [0,2] into 20 time units *TU_i_*,*i* = 0,…, 19, of 100 ms each; overall, there are a total of 320 units for all 16 channels. Let **U***^D^* be the discriminant unit map consisting of 320 units $u_{ij}^{D}$, where 0 ≤ *i* ≤ 15, 0 ≤ *j* ≤ 19. Each $u_{ij}^{D}$ indicates the number of elements that equal one in the *j*-th unit of the *i*-th row. In this scheme, the higher value of $u_{ij}^{D}$, the more important of a role that the *j*-th unit in the *i*-th row of map **M***^D^* plays. The above process is implemented as follows:

- Step 1: From the training data **x***_k_* = [*x_k_*_1_, ‥, *x_kn_*]*^T^*, *k* = 1, ‥, *N*, using R-LDA to obtain *n_f_* projection vectors $w_{l}^{R}$, *l* = 1‥n*_f_*.
- Step 2: For each projection vector $w_{l}^{R}$, construct the dimensional map $m_{l}^{R}={\left[m_{l1}^{R},\ldots ,m_{lJ}^{R}\right]}^{T}$, as follows:

(6) $$m_{li}^{R}=\left|w_{li}^{R}\right|.$$

- Step 3: Construct a discriminant information map **m***^D^* by summing $m_{l}^{R}$ for *l* = 1, …, *n_f_*:
  (7) $$m^{D}=\sum_{l=1}^{n_{f}}m_{l}^{R}$$
- Step 4: To effectively distinguish important parts based on the magnitude of $m_{i}^{D}$, we define an order vector **o** = [*o*_1_, …, *o_J_*]*^T^*. In the vector **o**, the *i*-th component *o_i_* indicates the order of the absolute value $\left|m_{i}^{D}\right|$, sorted in ascending order. For example, if *o_i_* is assigned the value (*n* − *k* + 1), then $m_{i}^{D}$ is the *k*-th largest value in $\left\{m_{i}^{D}|i=1,\ldots ,J\right\}$. Finally, the elements of the discriminant information map **m***^D^* are modified as follows:
  (8) $$\{\begin{array}{ll} m_{i}^{D}=1, & \text{if}\;o_{i}\geq n-N\\ m_{i}^{D}=0, & \text{otherwise}. \end{array}$$
  Here, **N** is denoted as the total number of selected pixels. In this scheme, if the element $m_{i}^{D}=1$, it means that the *i*-th pixel of **m***^D^* is considered to be important in this discriminant information map.
- Step 5: Transform the discriminant information map **m***^D^* into a 16 × 2000 matrix form **M***^D^*. Divide the overall time-horizon [0, 2] into 100-ms intervals, and the rearrange map **M***^D^* contains 16 channels, each separated into 20 time units. Count the number of elements *m_i_*'s that equal one in each unit.
- Step 6: Define the discriminant unit map $U^{D}=\left\{u_{ij}^{D}\right\}$, 0 ≤ *i* ≤ 15, 0 ≤ *j* ≤ 19, where $u_{ij}^{D}$ is the number of elements *m_i_*s that equal one in the *j*-th unit of the *i*-th row of **M***^D^*.

### 3.2. Pseudo Optimization of E-Nose Data Based on the Two-Dimensional Discriminant Map

The two-dimensional discriminant information map **U***^D^* defined in the previous section can be used to represent the rearranged discriminant information map **M***^D^* at the unit level. In map **U***^D^*, an element with value one indicates that its corresponding unit in **M***^D^* has high discriminant information.

High value elements in **U***^D^* are distributed heterogeneously. This means that they mainly concentrate on certain channels and time-horizon frames, rather than dispersing over the whole map. Thus, we only use elements of **U***^D^* with high distributions, *i.e.*, we only choose the elements in important channels and time-horizon frames. The pseudo optimization of e-nose data based on the two-dimensional discriminant information map **U***^D^* is implemented as follows:

- Step 1: Divide the two-dimensional discriminant information map **U***^D^* into two parts, important and unimportant information, using window **W** = {[*C_i_*, *C_j_*], [*TU_m_*, *TU_n_*]}, where [*C_i_*, *C_j_*] represents all of the channels from *i* to *j* and [*TU_m_*, *TU_n_*] is all of the time-horizon frames from *m* to *n*. Elements inside **W** containing all of the channels from channel *i* to *j* and all of the time-horizon frames from *m* to *n* are important, while the remaining elements are discarded because they are unimportant. To do this, we construct a new map, denoted by ${\text{U}}^{M}=\left\{u_{ij}^{M}\right\}$, 0 ≤ *i* ≤ 15, 0 ≤ *j* ≤ 19, from **U***^D^* as follows:
  (9) $$\{\begin{array}{ll} u_{ij}^{M}=u_{ij}^{D}, & \text{if}\;u_{ij}^{D}\in W\\ u_{ij}^{M}=0, & \text{otherwise}. \end{array}$$
- Step 2: From the modified two-dimensional map **U***^M^*, reconstruct the modified rearranged map **M***^M^* (16 × 2000 matrix). If $u_{ij}^{M}=0$, all data points in the *j*-th unit of the *i*-th row in **M***^M^* are set to zero. Otherwise, if $u_{ij}^{M}=1$, all elements in the corresponding position of **M***^M^* are set to the value of their equivalent elements in **M***^D^*.
- Step 3: Convert the modified rearranged map **M***^M^* into the modified one-dimensional discriminant information array **m***^M^*. This final map is used for the feature extraction and classification stage.

## 4. Experimental Results

In this section, we present the applications of the proposed method to the e-nose system described in [11]. Since the efficacy of the proposed region selection method is evaluated based on the comparison with previous works in [16,18], we apply our method to the same e-nose dataset for the implementation. The volatile organic compound (VOC) measurement data consisted of eight classes: acetone, benzene, cyclo-hexane, ethanol, heptane, methanol, propanol and toluene. The dataset contained 160 samples, and each sample (**x***_k_* ∈ ℝ^32000^) consisted of 32, 000 variables that were measured through 16 channels over 2000 time points. To evaluate the classification rates, we performed five-fold cross-validation [25] and computed the average value. There were 128 data samples in the training set and 32 data samples in the testing set.

**Figure 6.**
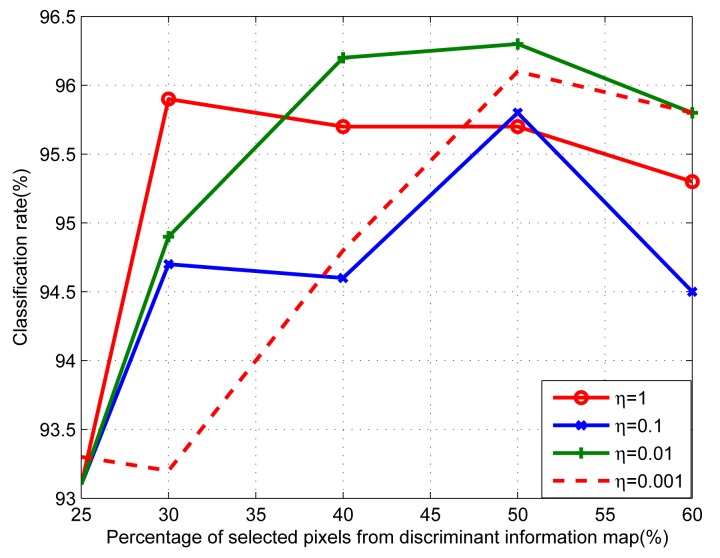
The classification rates with different *η* values.

For the experiment using the proposed method, we first found a suitable value of the regularization parameter *η* in the R-LDA equation. To do this, we first applied the R-LDA-based feature feedback mentioned in Subsection 2.3 to e-nose data for different values of *η*. For each value of *η*, we used a different number of selected value in the feature mask obtained using R-LDA. We compared the performances of all cases to determine the best value of *η*. Figure 6 shows the comparison of classification rates for various values of *η*. As depicted in Figure 6, the classification rate changes according to the value of *η*, and the number of selected elements in the feature mask **N** also changes. Based on these results, we chose *η* = 0.01 for our experiments.

### 4.1. Construction of Two-Dimensional Discriminant Information Map

To construct the one-dimensional discriminant information map **m***^D^*, we set *n_f_* = 3, because the sum of the three largest eigenvalues of $x_{l}^{L}$, *l* = 1, ‥, 3, accounted for approximately 99% of the total sum of the eigenvalues. Among the 32,000 elements of the discriminant information map **m***^D^*, we perform experiments using only 8000, 9600, 12,800, 16,000 and 19,200, according to 25%, 30%, 40%, 50% and 60%, respectively, of the highest values in map **m***^D^*. All of the selected elements were set to one, and the remaining elements were set to zero. By doing this, the discriminant information map **m***^D^* was divided into two parts: the important part with all 1 elements and the unimportant part with all 0 elements.

As mentioned in Section 3, in the rearranged discriminant information map **M***^D^*, we divided the time-horizon [0, 2] into periods of 100 ms and obtained 20 time units *TU_i_*, *i* = 0, .., 19; in other words, each channel was divided into 20 units. The discriminant information map **M***^D^* was represented at the unit level by introducing the two-dimensional discriminant information map **U***^D^*.

**Figure 7.**
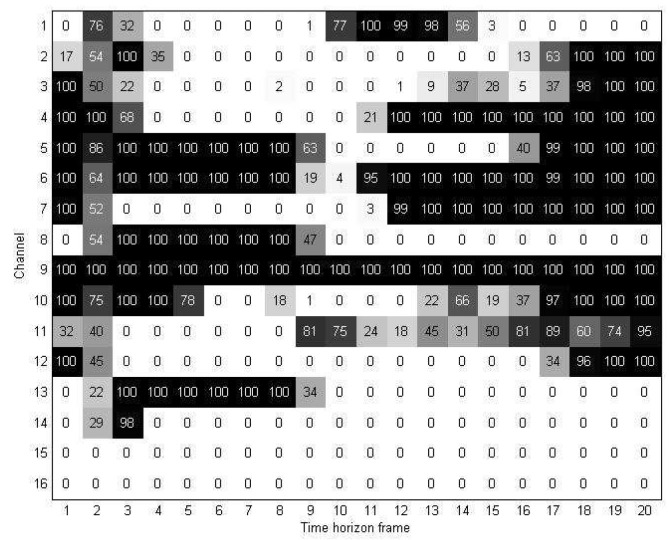
Two-dimensional discriminant information map.

Figure 7 shows an example of a two-dimensional discriminant information map **U***^D^* obtained from 128 training samples of the first experiment. As mentioned earlier, in **U***^D^*, each element $u_{ij}^{D}$ indicates the number of high elements in its corresponding location in the rearranged discriminant information map **M***^D^*. As a result, the higher the value of $u_{ij}^{D}$ in **U***^D^*, the more important of a role the *j*-th unit of the *i*-th channel plays in the rearranged discriminant information map **M***^D^*.

### 4.2. Region Selection Based on the Two-Dimensional Discriminant Information Map

**Figure 8.**
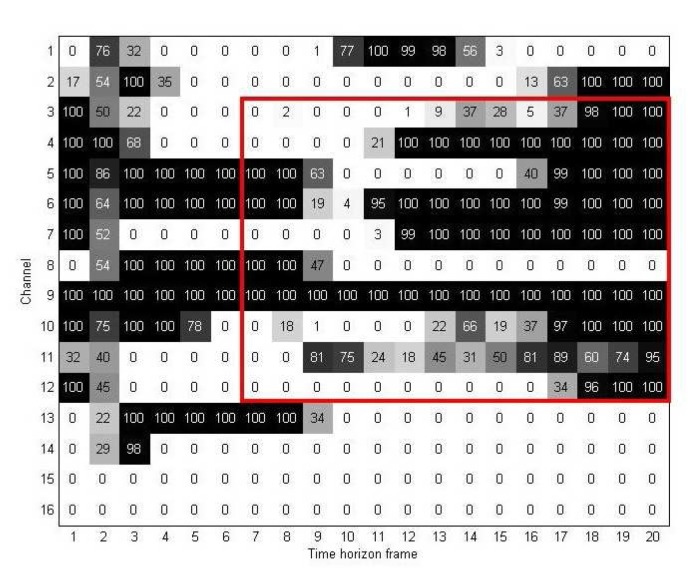
Discriminant information map **U***^D^* with region selection using window **W**.

**Figure 9.**
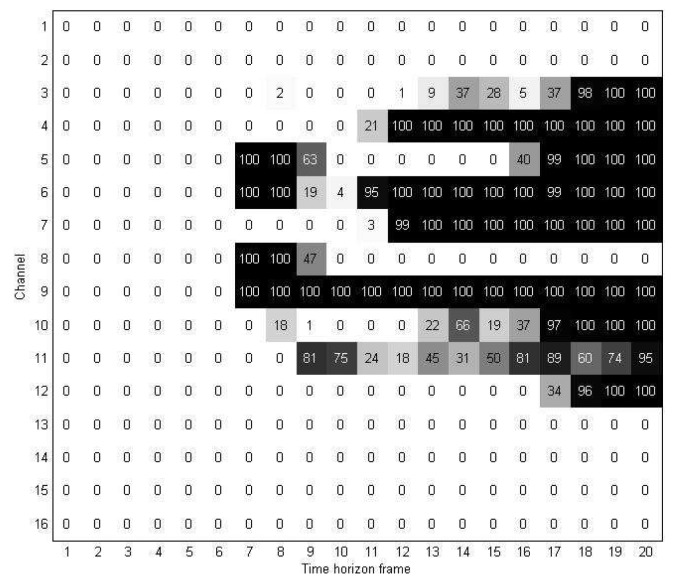
The two-dimensional discriminant information map **U**^M^.

For the region selection process, we first divide map **U***^D^* into two parts using window **W**. Figure 8 gives an example to illustrate how we used window **W** and two-dimensional discriminant information map **U***^D^* for the region selection method. In this figure, the vertical and horizontal edges of **W** indicate which channels and time-horizon frames are used to extract the important elements from **U***^D^*. Figure 9 shows the resulting map after the channel and time selections are applied. As mentioned in Section 3.2, we denote this new discriminant map as **U***^M^*.

In our experiment, for different values of **N**, the window **W** was determined heuristically by changing the selected region or selected channels and time frames. We compared all of the results to choose the best window **W** for each case. Note that for each map **U***^D^*, we use only one window **W** for the selection, *i.e.*, we extracted the elements of continuous channels, as well as time frames. Doing this made our method more practical and easier to implement for real-life applications.

### 4.3. Classification Using the Selected Region

In this section, we discuss the results from two experiments conducted to evaluate how our proposed method affects the classification rate. In the first experiment, we compared the classification rate when R-LDA-based feature feedback, with and without the region selection method, was used during the classification stage. In the second experiment, we compared our selection method with other selection methods, such as channel selection and time-horizon selection.

For each experiment, the variables of all samples in the training set were normalized using the mean and variance of the training set. The features used for classification were extracted using the region selection from the discriminant information map created by R-LDA. For the classification stage, the one nearest neighbor algorithm was used as a classifier, the same as in [16,18]. Since the proposed region selection method focuses on the data optimization problem at the feature extraction stage, a simple classifier, such as one nearest neighbor, is used at the classification stage to make it easier to evaluate the effects of our data selection method on the e-nose system performances. In both experiments, the distances between the pairs of samples were measured using the *l*_2_ norm.

Table 2 shows the results from the first experiment. We implemented the R-LDA-based feature feedback with and without the region selection for different values of **N**. For each value of **N**, we obtained a different two-dimensional discriminant information map **U***^D^*; this required using a different window **W** for region selection. The results in Table 2 clearly show the effects of region selection on the e-nose system. When more than 30% of the highest pixels in map **m***^D^* are selected, the recognition rate of the proposed selection method is always higher than that of the R-LDA-based feature feedback without any selection method. In the best case, the average recognition rate when data from regions containing three to 12 channels and seven to 20 time-horizon frames is 1% higher than that when all of the channels and time-horizons are considered.

Note that when using the region containing Channels 3 to 12 and time-horizon Frames 7 to 20, only 44% of the whole data is utilized, and the recognition rate improves by 1%. This suggests that the proposed method has the ability to enhance performance, specifically the processing time, memory size and recognition rate.

**Figure 10.**
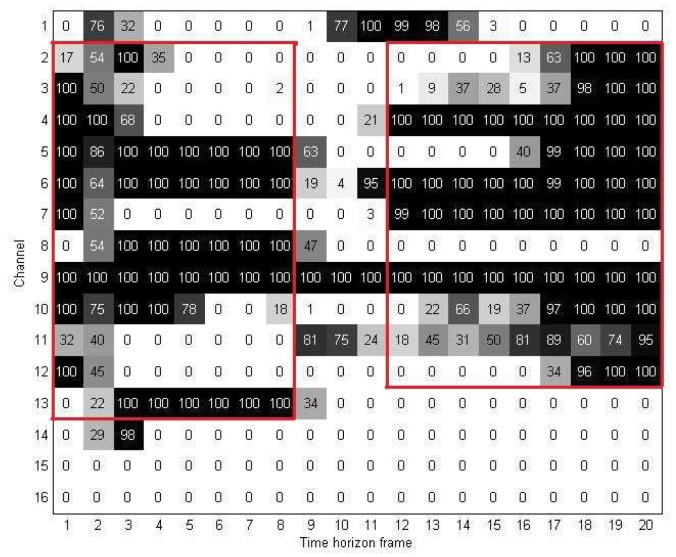
Utilizing two windows **W** for region selection.

Table 3 shows the results from the second experiment. In this experiment, we compared the effects of different methods applied to the e-nose data. When all of the elements in the discriminant information map (without any selection method) are used for classification, the R-LDA-based feature feedback obtains a higher classification rate than the PCA-LDA-based feature feedback. The classification rate improves considerably when a selection method is applied to the discriminant information map. By applying the region selection method, the classification rate not only improves, but reduces a large amount of usage data in the discriminant information map. The last row of Table 3 shows the result when two windows are used instead of one for region selection (shown in Figure 10). In this case, although the classification rate improves by 0.4%, the usage data are much higher than 17%, which are the usage data when only one window is used.

## 5. Conclusions

In this paper, we presented a region selection scheme applied to a vapor classification system using data from a portable e-nose sensor.

The high-dimensional input data from the e-nose sensor are highly redundant, caused by measurement noises, sensor errors and unimportant parts of the dataset. As a result, an analysis with the original input data is used, requiring an enormous amount of memory size, computation time and power consumption. Once the relative importance of data between channels and the time horizon is determined, we can efficiently extract important data for the classification process, while removing the redundant information and, hence, improving the performance of the classification system.

Consequently, we have proposed a region selection method of sensor data using feature feedback. First, we have created a two-dimensional discriminant information map by using R-LDA. Then, from the discriminant information map, we extracted important data by merging useful channels and time-horizon frames. With the region selection method, we can reduce the processing time, required memory, power consumption, and so on. Furthermore, we can improve the performance of classification by eliminating the redundant data. From the experiment for the e-nose system, we have shown that the performance of the classification can be improved in the sense of the classification rate, data processing time and memory size.

As future work, we require a more systematic algorithm in order to merge the selected channels and time horizon frames. In addition, a more complex data set for the experiments with the proposed method is also the subject of future work.

## Figures and Tables

**Table 1.** Characteristics of PCA, LDA and regularized linear discriminant analysis (R-LDA).

**Method**	**Scatter Matrix Used**	**Objective Function**
PCA	$S_{T}=\sum_{i=1}^{C}\left(x_{i}-\mu \right){\left(x_{i}-\mu \right)}^{T}$	*W^P^* arg max*_W_* |*W^T^ S_T_W*|
LDA	$\begin{array}{c} S_{B}=\sum_{i=1}^{C}N_{i}\left(\mu_{i}-\mu \right){\left(\mu_{i}-\mu \right)}^{T}\\ S_{W}=\sum_{i=1}^{c}\sum_{x_{k}\in c_{i}}\left(x_{k}-\mu_{i}\right){\left(x_{k}-\mu_{i}\right)}^{T} \end{array}$	$W^{L}\arg\max_{W}\frac{\left|W^{T}S_{B}W\right|}{\left|W^{T}S_{W}W\right|}$
RLDA	$\begin{array}{c} S_{B}=\sum_{i=1}^{C}N_{i}\left(\mu_{i}-\mu \right){\left(\mu_{i}-\mu \right)}^{T}\\ S_{W}=\sum_{i=1}^{c}\sum_{x_{k}\in c_{i}}\left(x_{k}-\mu_{i}\right){\left(x_{k}-\mu_{i}\right)}^{T} \end{array}$	$W^{R}\arg\max_{W}\frac{\left|W^{T}S_{B}W\right|}{\left|\eta \left(W^{T}S_{B}W\right)+W^{T}S_{W}W\right|}$

*μ*: mean of the whole training samples; *μ_i_*: mean of the samples belonging to class *C_i_* that has *N_i_* ; *η*: regularization parameter (0 ≤ *η* ≤ 1).

**Table 2.** Each classification rate from the five-fold cross-validation for selected channels and time-horizon (%).

	**Channel and Time Horizon**	**Feature (1,2, 5−7)**					**Average**
N = 8000 (25%)	All data [29]	84.4	96.9	90.6	90.6	90.6	90.6
	[*C*_3_, *C*_10_], [*TU*_12_, *TU*_20_]	65.6	87.5	84.4	90.6	90.6	84.4
N = 9600 (30%)	All data [29]	72.5	93.1	97.5	97.5	97.5	93.2
	[*C*_3_, *C*_10_], [*TU*_12_, *TU*_20_]	76.9	93.1	98.1	98.1	98.1	94.1
N = 12800 (40%)	All data [29]	78.8	97.5	97.5	97.5	97.5	94.6
	[*C*_3_, *C*_12_], [*TU*_7_, *TU*_20_]	83.8	99.4	99.4	99.4	99.4	97.2
N = 16000 (50%)	All data [29]	86.3	96.3	98.1	98.1	98.1	96.2
	[*C*_1_, *C*_12_], [*TU*_9_, *TU*_20_]	86.3	96.9	98.8	98.8	98.8	96.3
N = 19200 (60%)	All data [29]	83.1	96.9	98.1	98.1	98.1	95.8
	[*C*_1_, *C*_12_], [*TU*_9_, *TU*_20_]	80	97.5	98.8	99.4	99.4	96

**Table 3.** Average classification rates of different selection methods (%).

**Methods Feature**	**1**	**2**	**5**	**6**	**7**	**Average**	**Data Size Used (%)**
PCA + LDA, All [18]	87.6	95	97.5	97.5	97.5	95.3	100

PCA + LDA, Channel selection [16]	91.1	98.1	98.8	98.8	98.8	97.8	56
Channel {1, 8, 2, 14, 3, 5, 16, 6, 9}							

PCA + LDA, Time horizon selection [18]	85.6	96.9	99.4	99.4	99.4	96.9	55
Time horizon {*TU*_9_ − *TU*_19_}							

R-LDA, All [29]	86.3	96.7	98.8	98.8	98.8	96.3	100

R-LDA, Channel and time selection	83.8	99.4	99.4	99.4	99.4	97.2	44
Channel and time {[*C*_3_ − *C*_12_], [*TU*_7_ − *TU*_20_]}							

R-LDA, Channel and time selection	87.9	99.6	99.6	99.6	99.6	97.6	61
Channel and time {[*C*_3_ − *C*_12_], [*TU*_1_ − *TU*_8_]}, {[*C*_3_ − *C*_12_], [*TU*_12_ − *TU*_20_]}							
